# Pioneering Neglected Disease Research in Southern Mexico at the “Dr. Hideyo Noguchi” Regional Research Center

**DOI:** 10.1371/journal.pntd.0002530

**Published:** 2013-11-21

**Authors:** Eric Dumonteil, Miguel Rosado-Vallado, Jorge E. Zavala-Castro

**Affiliations:** 1 Laboratorio de Parasitología, Centro de Investigaciones Regionales “Dr. Hideyo Noguchi,” Universidad Autónoma de Yucatán, Mérida, Yucatán, Mexico; 2 Director Centro de Investigaciones Regionales “Dr. Hideyo Noguchi,” Universidad Autónoma de Yucatán, Mérida, Yucatán, Mexico; Yale School of Public Health, United States of America

## Historical Perspective

The Autonomous University of Yucatan is a major public university in Mexico, and the largest institution for superior education in the southern part of the country, with 772 academics attending over 20,000 students. It offers 43 undergraduate and 53 graduate programs in all areas of knowledge.

On October 12, 1975, Dr. Alberto Rosado G. Cantón, rector of the University at this time, created the Biomedical Research Center to provide the institution with a leading facility to perform scientific medical research. The Center was named in honor of Dr. Hideyo Noguchi, the famous Japanese scientist who worked on yellow fever in the region from 1918–1920 and received a Doctorate Honoris Causa from the School of Medicine of the University in 1920. The Research Center initiated its activities with three departments: Tropical Pathology, headed by Dr. Jorge Zavala-Velazquez; Reproductive Health, headed by Dr. Thelma Cetina-Canto; and Physiology, headed by Dr. Heriberto Arcila-Herrera. In 1976, the Center was strengthened by the incorporation of several researchers in social sciences, and renamed “Dr. Hideyo Noguchi” Regional Research Center. In 1987, it became organized in a Social Science and a Biomedical Unit, to accommodate wider research interests encompassing virology, microbiology, parasitology, reproductive health, human genetics, hematology, social medicine, neurosciences, regional development, Mayan identity and culture, and political and social processes.

The department of Tropical Pathology initially pioneered some key research in Mexico and in the region in the area of tropical medicine and several neglected diseases. It also served as a seed for the training of several young researchers who contributed to the growth of the Research Center by becoming group leaders themselves in the following years, including Dr. Andrade-Narvaez, Dr. Puerto-Manzano, Dr. Farfan-Ale, and Dr. Barrera-Perez, and more recently Dr. Zavala-Castro, Dr. Garcia-Miss, Dr. Acosta-Vianna, and Dr. Vargas-González, among others.

## The “Dr. Hideyo Noguchi” Regional Research Center Today

Today, the Center has established itself as a major research facility in southern Mexico, hosting 66 researchers and 44 research technicians. Its mission is to develop research in health, disease, social sciences, and humanities of regional, national, and international relevance. In 2012, the Biomedical Unit alone received a total of about US$1,500,000 in external research grants from a number of national and international funding agencies. The Center also plays a major role in the training of qualified human resources in health and social sciences. Over the years, 336 undergraduate and 73 graduate students have developed their theses in its laboratories, and many more received training during shorter internships. The staff of the Center also contributes to formal teaching in many of the academic programs of the University, thus bringing cutting-edge knowledge and research to students in biology, chemistry, human and veterinary medicine, mathematics, and social sciences, among others. The creation of a M.Sc./Ph.D. program in Health Sciences in 2009 was a major milestone toward increased scientific capacity building.

**Figure 1:**
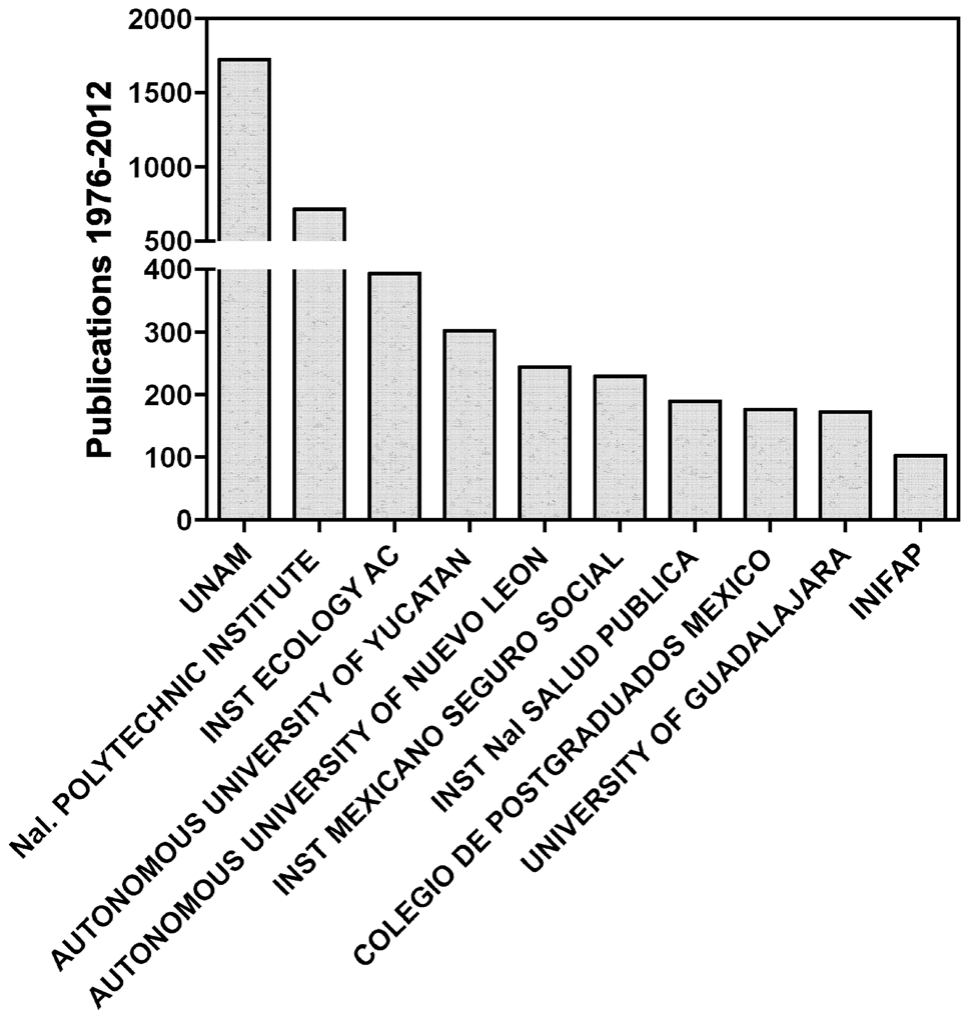
Scientific production of leading institutions in Mexico on neglected tropical diseases. Data are from the Web of Science for years 1976–2012, from the following research areas: parasitology, infectious diseases, tropical medicine, and entomology.

Importantly, infectious and tropical diseases have remained at the heart of its research and teaching activities, and the “Hideyo Noguchi” Regional Research Center has contributed significantly to the ranking of the Autonomous University of Yucatan as one of the most productive institutions in the area of tropical medicine and neglected disease research in Mexico (Figure 1). The university is indeed ranked in fourth place in terms of scientific production in this area, only following huge national institutions such as the National Autonomous University of Mexico with over 37,000 academics, the National Polytechnics Institute with over 16,000 researchers, and the Institute of Ecology with about 100 researchers. Given the small size and the limited infrastructure of the Center within the university system, this achievement is a remarkable feat and a real testimony of the dedication of the academics in this field.

## Major Scientific Achievements

Throughout the years, the Center has contributed major advances in the understanding and control of several tropical diseases caused by viruses (dengue, West Nile virus, and rotavirus), bacteria (*Salmonella*, *Rickettsia*, *Leptospira*, *Toxoplasma*, and *E. coli*), and parasites (*Trypanosoma cruzi* and *Leishmania*), as evidenced by some highly cited studies.

### Dengue and Other Flaviviruses

These viruses have been studied at the Center since 1983, following the first detection of dengue in Mexico in 1978 and in the Yucatan region in 1979. In 2012, there were 44,607 confirmed dengue cases in Mexico, and the state of Yucatan was one of the hot-spots for dengue transmission, with 5,654 confirmed cases according to the Ministry of Health. Key studies led by Dr. Farfan-Ale and Dr. Loroño-Pino in collaboration with Colorado State University have focused on the molecular epidemiology and evolution of dengue virus, allowing for the description of the circulation of all four dengue serotypes in southern Mexico over the past 20 years [1], as well as mixed infection with more than one serotype in both viremic serum samples and mosquitoes [2]. Following the introduction of West Nile virus to North America in 1999, active surveillance for evidence of infection with this virus in migratory and resident birds was established in March 2000. This led to the first evidence that West Nile virus was indeed circulating in birds from the Yucatan peninsula [3]. Current research includes further monitoring and surveillance of circulating flaviviruses, as well as vector control for the reduction of dengue transmission.

### Rickettsiosis

While northern states of Mexico have been known to be endemic for Rocky Mountain spotted fever since the 1940s, it is only recently that febrile patients who are sometimes diagnosed as negative for dengue virus have been investigated further for an alternative diagnosis. This led to the identification of the first cases of rickettsiosis in the region by the group of Dr. Zavala-Velazquez [4], followed by the molecular identification of *Rickettsia felis*, *R. akari*, *R. rickettsii*, and *R. typhi* in different hosts and vectors by the group of Dr. Zavala-Castro [5], in collaboration with the University of Texas Medical Branch in Galveston. These findings opened the way to the search for the different transmission cycles of *Rickettsia* spp. in southern Mexico, including the identification of hosts, reservoirs, and vector species, with the general aim of understanding the potential risks and epidemiological importance of rickettsiosis. Further efforts are also directed toward vaccine development.

### Leishmaniasis

Leishmaniasis has been studied at the Research Center since 1982 through a series of integrated and multidisciplinary approaches investigating epidemiological, clinical, immunological, parasitological, entomological, and socioeconomic aspects of the disease. Pioneering studies by the group of Dr. Andrade-Narvaez in collaboration with the University of Texas have focused on the characterization of the immune response of patients, in an attempt to understand the changes in cytokine balance involved in the different clinical presentations of infections by *Leishmania (L.) mexicana* [6], [7]. More recent work focuses on the development of *Peromyscus yucatanicus*, a wild mouse from the region, as a unique animal model mimicking human disease, to further unravel the mechanisms of disease progression [8]. A vaccine candidate against several species of *Leishmania* has also been identified and is being further developed by the group of Dr. Dumonteil [9].

### Chagas Disease

The study of Chagas disease has been at the heart of the Center since its creation in 1975, with the first eco-epidemiological studies performed in the region by Dr. Zavala-Velazquez. With a seroprevalence of 1–5% in the region, and a national average of at least 1–2%, Chagas disease remains nonetheless a controversial health problem in Mexico, with limited epidemiological surveillance and no national vector control program. Key research by the group of Dr. Dumonteil, in collaboration with the University of Perpignan, France, has focused on *Triatoma dimidiata*, the main vector in the region, to develop and adapt vector control interventions to the ecology and biology of this species that is able to seasonally invade houses [10], [11]. With the recognition that many other triatomine species behave similarly, the integrative knowledge gained can help improve the control of many other invasive triatomine species [12]. Following a series of promising preclinical studies [13], Dr. Dumonteil's group is also part of the first public-private partnership aiming at developing a vaccine against Chagas disease [14], which opens new avenues for improved control of this neglected disease. This partnership includes BIRMEX, CINVESTAV, Baylor College of Medicine, the Sabin Vaccine Institute, and the National Institutes of Health as its main partners.

## Looking Toward the Future

A major challenge for the Center is to increase its social impact and promote the translation of the knowledge generated into public health policies and research priorities. Several researchers serve as advisers to local and national health authorities, and their expertise in the diagnosis of several pathogens such as dengue, West Nile virus, influenza, *Riskettsia* spp., and *Leishmania* is strengthening epidemiological surveillance. Nonetheless, published research needs to better contribute to the shaping of regional and national health policies and research priorities. Another challenge is to consolidate the many years of growth of the Research Center by fostering the continued incorporation of high-level researchers and the training of a new generation of health professionals. Finally, an important lesson learned, and a key factor contributing to the growth of the different research groups, has been the fundamental support of an impressive network of collaborators from national and international institutions (over 90 collaborating institutions). It is hoped that further expansion of this network through new collaborations will promote further growth and impact of the Center. In this situation, the “Dr. Hideyo Noguchi” Regional Research Center is emerging as a major reference center for research and training in tropical and neglected diseases, with a growing influence at the national, regional, and international level.
